# Improving public transportation systems with self-organization: A headway-based model and regulation of passenger alighting and boarding

**DOI:** 10.1371/journal.pone.0190100

**Published:** 2017-12-29

**Authors:** Gustavo Carreón, Carlos Gershenson, Luis A. Pineda

**Affiliations:** 1 Posgrado en Ciencia e Ingeniería de la Computación, Universidad Nacional Autónoma de México, Ciudad Universitaria, CDMX, México; 2 Centro de Ciencias de la Complejidad, Universidad Nacional Autónoma de México, Ciudad Universitaria, CDMX, México; 3 Computer Science Department, Instituto de Investigaciones en Matemáticas Aplicadas y en Sistemas, Universidad Nacional Autónoma de México, Ciudad Universitaria, CDMX, México; 4 SENSEable City Lab, Massachusetts Institute of Technology, Cambridge, Massachusetts, United States of America; 5 ITMO University, Saint Petersburg, Russian Federation; Beihang University, CHINA

## Abstract

The equal headway instability—the fact that a configuration with regular time intervals between vehicles tends to be volatile—is a common regulation problem in public transportation systems. An unsatisfactory regulation results in low efficiency and possible collapses of the service. Computational simulations have shown that self-organizing methods can regulate the headway adaptively beyond the theoretical optimum. In this work, we develop a computer simulation for metro systems fed with real data from the Mexico City Metro to test the current regulatory method with a novel self-organizing approach. The current model considers overall system’s data such as minimum and maximum waiting times at stations, while the self-organizing method regulates the headway in a decentralized manner using local information such as the passenger’s inflow and the positions of neighboring trains. The simulation shows that the self-organizing method improves the performance over the current one as it adapts to environmental changes at the timescale they occur. The correlation between the simulation of the current model and empirical observations carried out in the Mexico City Metro provides a base to calculate the expected performance of the self-organizing method in case it is implemented in the real system. We also performed a pilot study at the Balderas station to regulate the alighting and boarding of passengers through guide signs on platforms. The analysis of empirical data shows a delay reduction of the waiting time of trains at stations. Finally, we provide recommendations to improve public transportation systems.

## Introduction

There are 204 Metro systems in the world used by 112 million passengers every day [1]. In particular, the Mexico City Metro (MXM) serves approximately 5.5 million users each day and is integrated by 12 lines [2]. Public transportation systems (PTS) are conformed by an heterogeneous group of elements such as passengers, trains, tracks, stations and traffic lights. A computer model is useful to consider relations between their elements and for expressing the most representative variables and studying the effect of interactions [3–5]. Computer simulations based on real databases can replicate the dynamics of the system in different conditions and at the same time provide the capacity to test appropriate regulatory methods at different scales [6].

PTS work with a set of rules and procedures described in operation manuals. In many cases, systems are automatized or semi-automatized; sometimes, the operation is regulated manually. Moreira-Matias [7] provides a survey to improve the operational control and planning using automatic vehicle location and gives formal definitions in this area. For instance, in the MXM, a centralized system is used to monitor the locations of the trains using rail sensors, GPS is not available.

The application of a set of operating rules, the decision-making based on the system’s performance, and the behavior of users create the global dynamics of the system, which can be monitored by taking into account the most significant processes such as the time interval between trains, also known as the *headway*, alighting and boarding time of passengers and the waiting time of trains at stations. The MXM is considered a high frequency transit line due the small values of the headways: about two or three minutes under normal conditions.

In particular, the control of the headway is a fundamental problem in the regulation of the PTS [8], which can efficiently serve when trains arrive at stations according to regular time intervals [9]; however, a configuration of equal headways, although desirable, is intrinsically unstable [10, 11]. The equal headway instability is triggered by trains moving faster or slower than expected and the rapid change of the passenger’s demand [12]; this result in a “bunching” phenomenon [13]. In the MXM, a stop-skipping scheme is applied in overcrowded situations, *i.e.*, an empty train is sent from the dispatching terminal to the saturated station directly to reduce the passenger overload and maintain a regular headway. For this, a previous schedule planning is established and human operational intervention is required. An optimized approach using Genetic Algorithms to applied stop-skipping scheme has been proposed [14] and an online learning approach using real-time information [15], while predictive models using support vector machines [16] and transit smart card data [17], have been proposed to reduce and eliminate bus bunching. A factor to promote the headway instability is the overcrowding phenomenon; Li, *et al.* [18] proposed a method to predict the short-term passenger flow and contribute to crowd control measures.

The last approaches try to predict and face the stochastic dynamics of PTS. Nevertheless, some of the proposed improvements require a significant amount of resources supported by a suitable infrastructure.

This motivates the interest to regulate PTS from an adaptive perspective, trying to maintain and even recover equal headways. For instance Bartholdi and Eisenstein [13], use a self-equalized schema to generate a natural headway; Liang, *et al.* [19] propose a self-adaptive method to equalize headway by using local information to introduce a little slack time in a single line automatically; Daganzo [11] uses an adaptive control with information of the headway in real time to determines bus holding times; and Daganzo and Pilachowski [20] use cooperation between trains to adjust their cruising speed. One way to make the headway stable is using adaptive [10] or self-organizing methods [21]; in these previous works, we proposed regulatory methods to conserve and restore headways using abstract simulations with discrete time and space and normalized variables such as speed and passenger influx; it was shown that a self-organizing method can adjust waiting time intervals of trains in stations considering only local information; if the delay occurs in an inter-station segment, the self-organizing method can correct the headway when the train arrives at the station. These methods can achieve supra-optimal performance due to the slower-is-faster effect [22]: passengers may wait more time at stations, but once on trains, most probably they will reach their destination faster, as vehicles do not try to keep strict equal headways.

**Fig 1:**
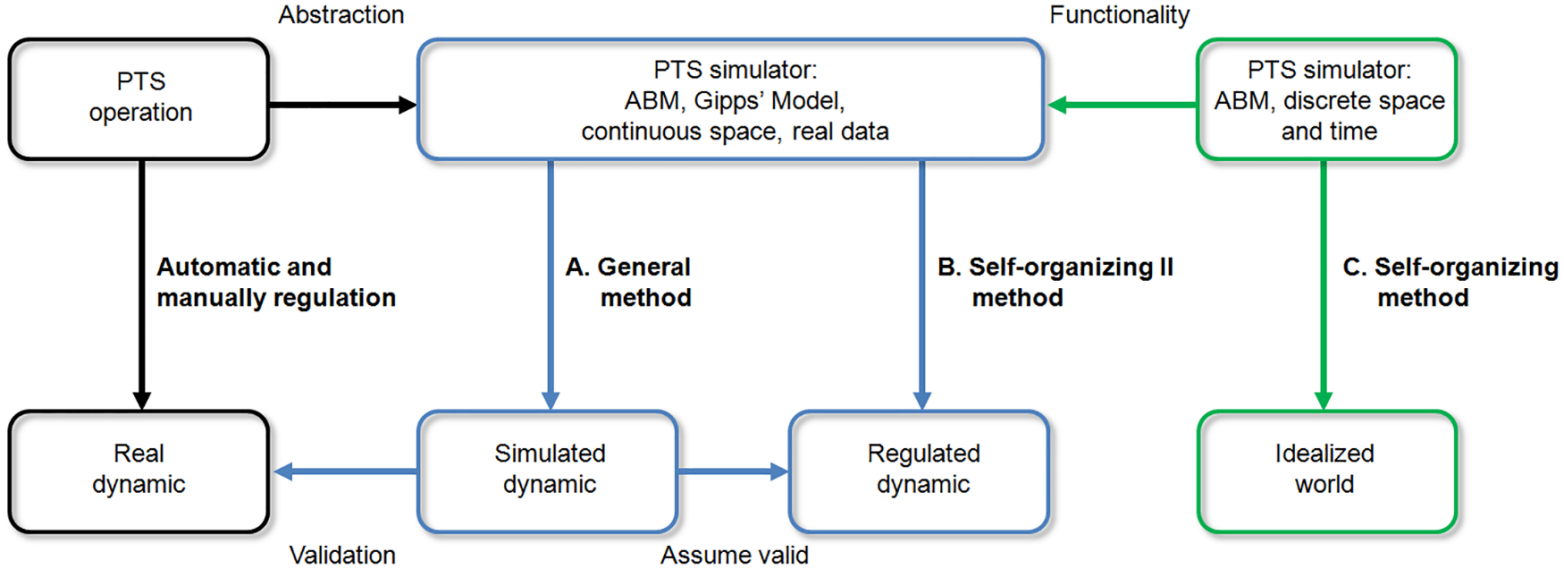
Relationship between methods (A) General, (B) Self-organizing II and (C) Self-organizing. This flowchart represents the relation between the methods (blue), real data (black) and an idealized abstract scenario (green).

This paper presents two regulatory headway control methods using the alighting and boarding time of passenger as a common basis. We refer to the first as the General Method (GM) (Fig 1A), which models the current dynamics of the headway of MXM through a minimum and maximum waiting time of trains at stations and a delay factor caused by the inflow and behavior of passengers in static and global context. The second is the Self-Organizing II Method (SOM-II) (Fig 1B) which is based on a previous self-organizing method [21] (Fig 1C) to regulate in an adaptive, local and decentralized manner the headway by using an *antipheromone* concept and the estimated time of arrival of the next train with a suitable balance of the variables to achieve self-organization.

The SOM-II may reduce the headway to a supra-optimal value as stipulated by inequality of Eq 1, as it adapts to the local flow of passengers and has the capacity to adapt the headway saving time in the passenger’s travel by avoiding vehicle idling.

$$\begin{array}{c} headway_{SOM-II}⩽headway_{optimal}⩽headway_{real} \end{array}$$ (1)

A computer simulation was developed to test the performance of both regulatory methods. Agent-based modeling (ABM) [23] and the NetLogo programming environment [24] were used. The reader can download the source code at https://github.com/gcarreon/pts-simulator.

Current data of the MXM Line 1 (pink) was used to feed the parameters to the computer simulation. This information was obtained empirically through recording and analyzing videos manually processed. The computer simulation considers three general processes: 1. dynamics of passengers, 2. train flow, and 3. regulatory methods.

Peter Gipps’ vehicular car-following model [25] was adapted to characterize the train flow, as detailed below. This model was extended with a sensor to identify agents in the line of vision to modify the behavior of the trains suitably. Also, a safe distance between trains was defined and autonomous braking through a “phantom object” strategy [26] was implemented.

The computer simulation supports two general conclusions: 1. the GM (Fig 1A) shows a high correlation with the observed dynamic of the headway in the MXM; and, 2. the SOM-II (Fig 1B) has a better performance than the GM, since it maintains regular headways in normal circumstances and restores them in the presence of perturbations. These results suggest that the improvement obtained with the SOM-II method in the simulation could be also observed in real systems if implemented, for which technology is available. Details of the methodology are presented. The Results section show the efficiency of the regulatory methods with its validation, followed by a Discussion. The description of an experiment pilot perform in MXM to regulate the alighting and boarding of passengers is described. Recommendations to improve PTS are finally presented.

## Methods

**Fig 2:**
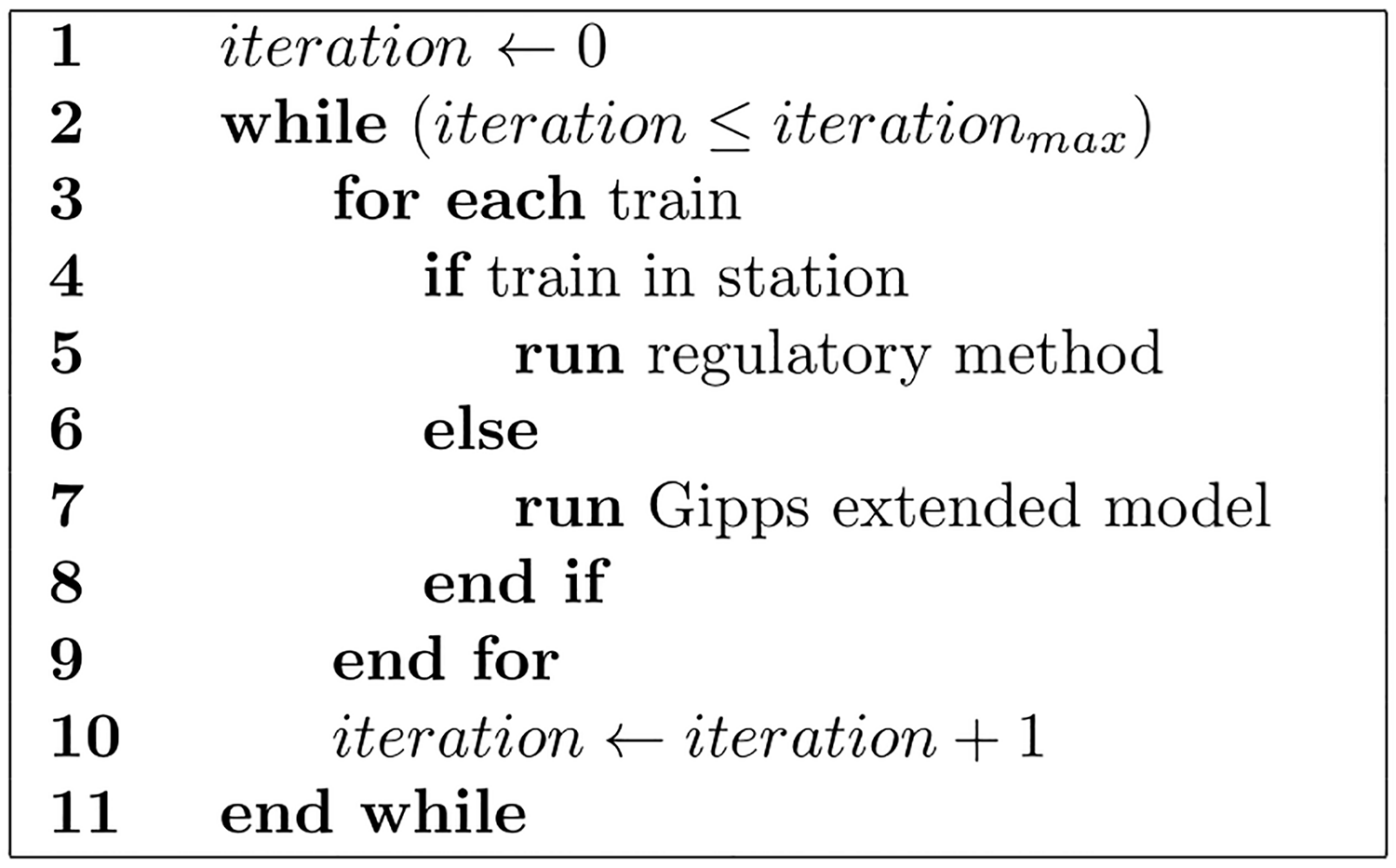
Simulator’s main control pseudocode.

Our computational methods consist of two parts: the first is the method to regulate the dynamics for each train at the station, which has two modalities: 1. The GM, and 2. the SOM-II. The second part is an extension of Gipps’ model to characterize the dynamics of the train’s flow and the interaction with agents in the environment. Fig 2 shows the simulator’s main control pseudocode.

**Fig 3:**
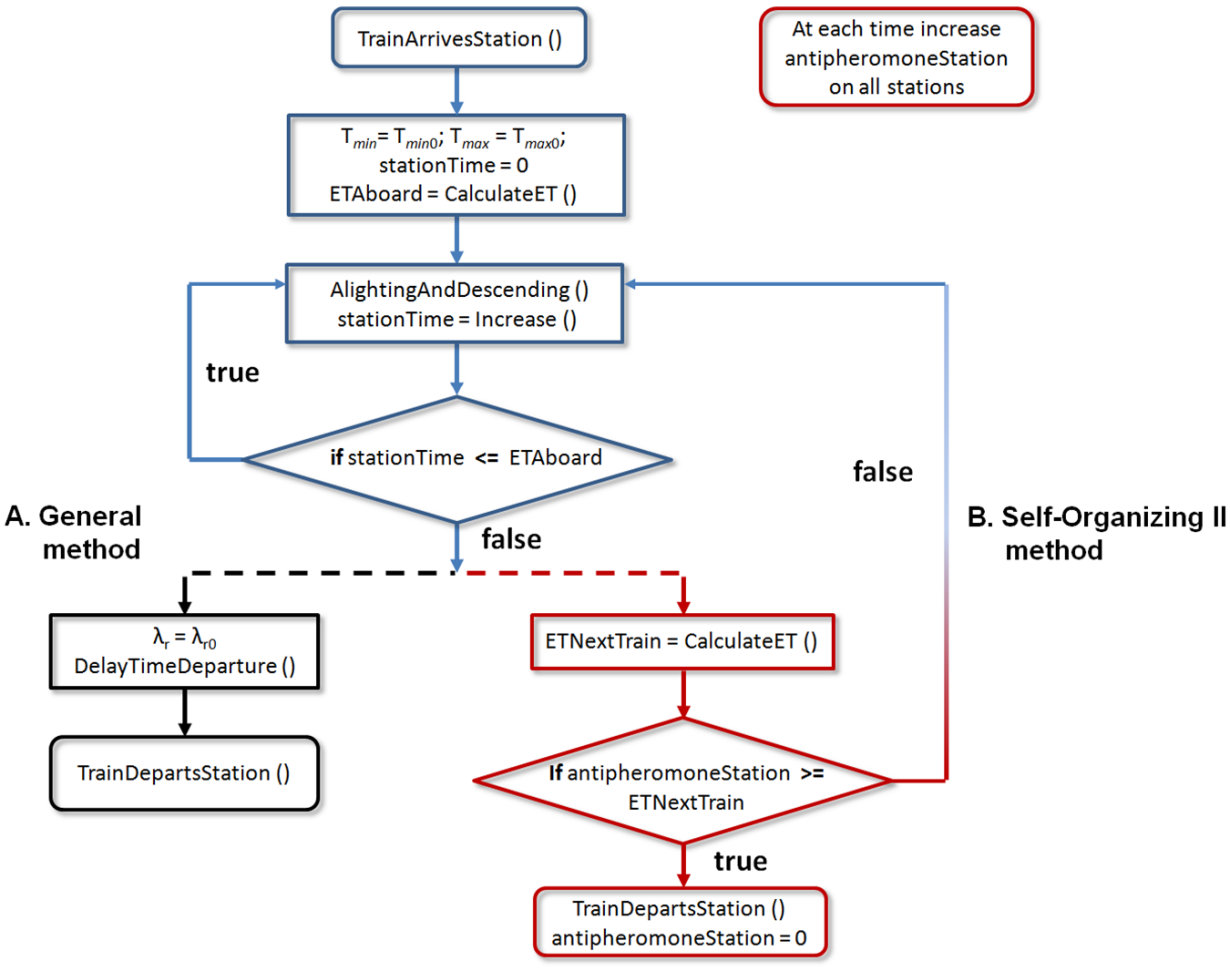
General flow diagram. (A) General method (black). (B) Self-Organizing II method (red). Common procedures are shown in blue. See text for explanation.

Both regulatory methods share the process of arrival of the train and the alighting and boarding of passengers procedure as illustrated in the upper side (in blue) in Fig 3.

This block starts with the arrival of the train at the station and stops due to a traffic light in red (all trains stop at each station). Each time that a new passenger is added to the simulation, a new registry is created with her origin and destination station following a probability distribution according to the real average’s passenger flow data from each station. This journey is calculated a priori based on this empirical information.

Each train has a control vector which records the path of the passengers. When a train arrives at a station; passengers who have ended their journey are identified and prepare for alighting. The estimated time for alighting and boarding is calculated in each station, *ETAboard*, from the number of passengers that alight and those who are in the platform to aboard; the total number of passengers are divided by four to model the four doors of the wagon. *ETAboard* is delimited by the minimum and maximum waiting time in the station, *T*_*min*_ ≤ *ETAboard* ≤ *T*_*max*_; these boundaries are set globally in the system with values empirically obtained. For instance, when there are no passengers to alight or board, the train will remain in the station time *T*_*min*_. Alternatively, if an overcrowded situation occurs, the train will stay until the maximum time *T*_*max*_ is reached. Alighting and boarding of passengers has a rate of one passenger per second; for example, if at a station there are 50 passengers alighting and 30 passengers boarding, then the train will wait 80/4 = 20 seconds (30 ticks). The cycle concludes when the estimated time *ETAboard* is reached. The train departure is determined by the regulation method, either GM (left side) or SOM-II (right side) (Fig 3A and 3B).

### General method

The dwell time is defined as the sum of the alighting and boarding of passengers at the *station*_*k*_ using a constant rate [12] and a delay factor modeled with a Poisson distribution with mean *λ*_*r*_, which represents the delay of the boarding process and potential crowding effects. Other studies considered the fare payment method [27] and automatic passenger counting and vehicle location data [28], but in MXM the payment method is at the station’s entrance and automatic passenger counting is not available.

When the alighting and boarding process concludes, a delay factor *λ*_*r*_ is applied as defined in the lower branch (in black), on the left in Fig 3A. The *DelayTimeDeparture* procedure delays the departure based on a Poisson distribution with mean *λ*_*r*_ seconds. The *TrainDepartsStation* function changes the traffic light to green so the train leaves the station. Subsequently, the traffic light changes to red to receive the next train. The previous section showed that the dynamic’ MXM could be modeled with appropriate parameter values *T*_*min*_, *T*_*max*_ y *λ*_*r*_.

### Self-organizing II method

**Fig 4:**
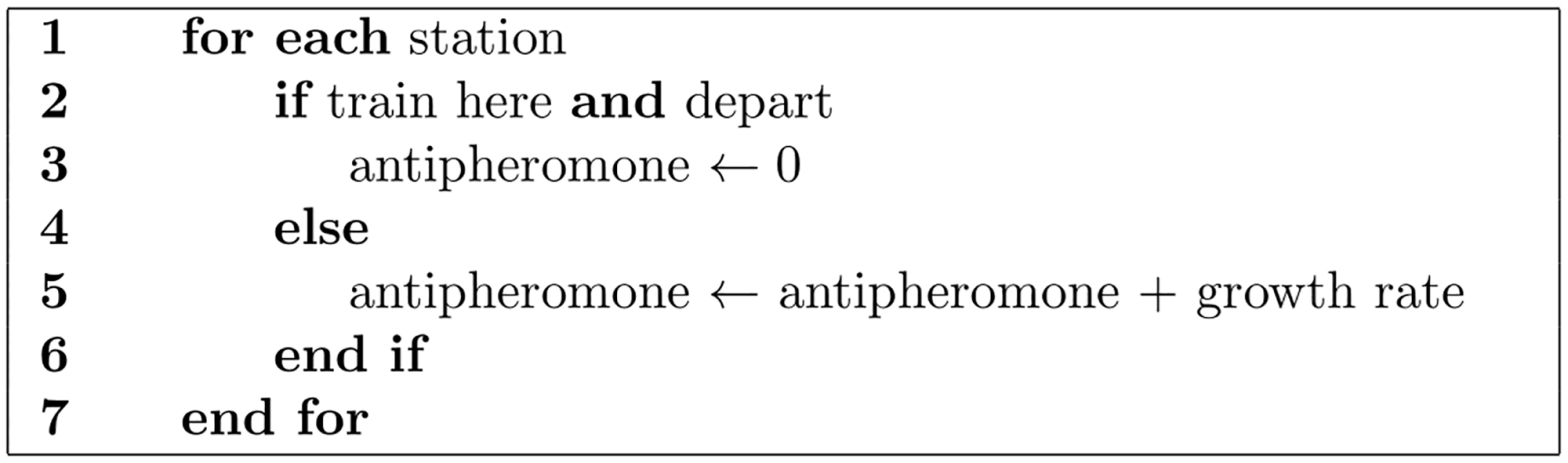
Pseudocode of antipheromone update.

The Self-organizing II method regulates by adapting the time of the train in the station. This method uses the anti-pheromone concept [21], evoking the communication of social insects through the environment [29, 30]. A variable assigned to each station represents the anti-pheromone; its value is zero once the simulation starts and increases at a growth rate of 2/3 per iteration. It is initialized to zero each time a train leaves the station. This value represents the elapsed time since the previous train left. This process is included in the right-upper red block in Fig 3. The procedure to calculate its value is shown in the pseudocode of Fig 4.

**Fig 5:**
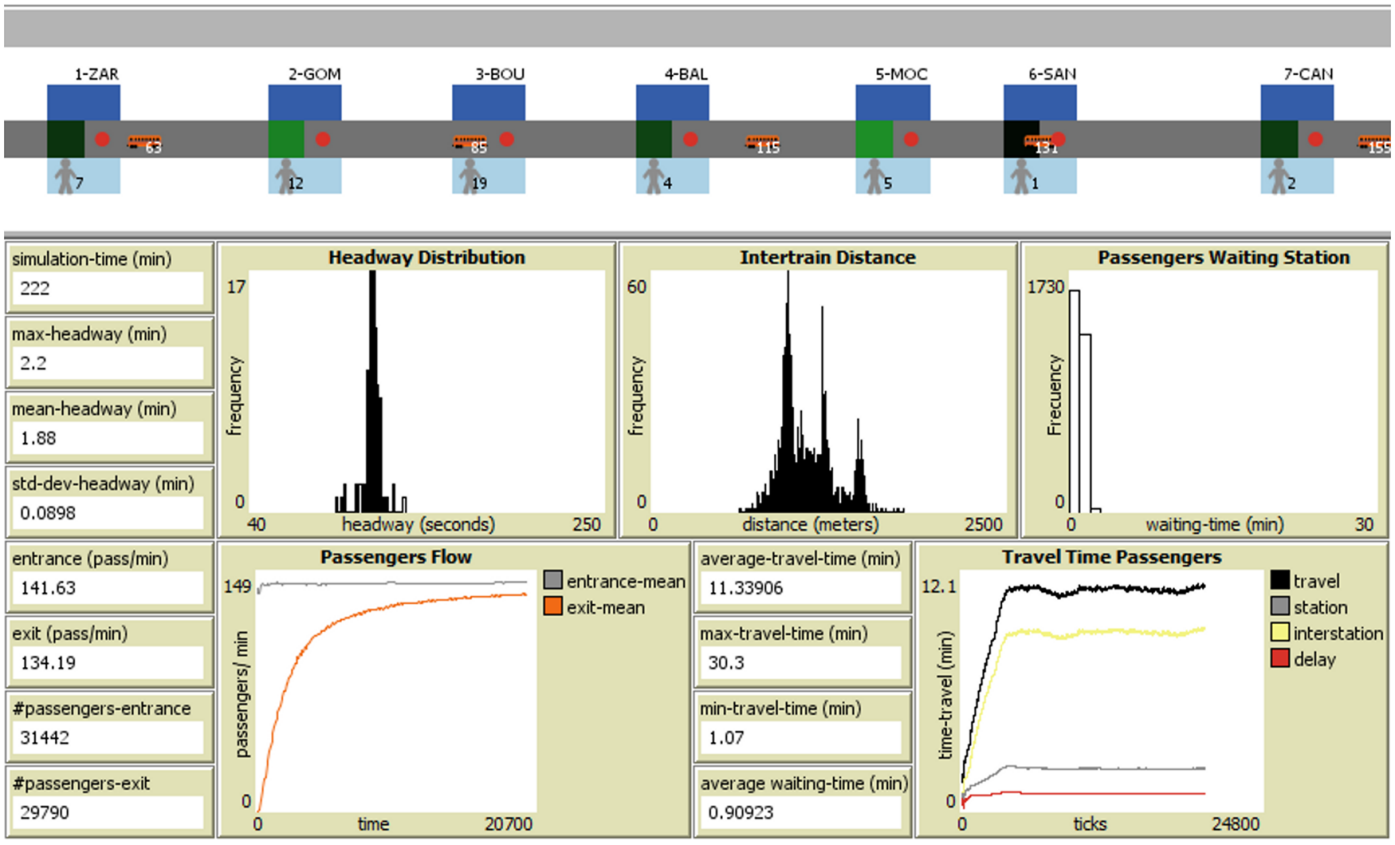
Screenshot of the simulation using the Self-Organizing Method II for 20,000 iterations. The characteristics of the Line 1 of the MXM are considered. The histogram of the headway frequency shows a mean value around 1.88 min and a small standard deviation, 0.0898 min, which reflects a global stability on the system. Regarding the average travel time of passengers, these are stable and remain constant. The intensity of the green color at stations is proportional to the antipheromone concentration.

The mechanism to determine if the train departs or stays is the result of the comparison between the antipheromone value and the estimated time of arrival of the next train, *ETNextTrain*; see the low part (in red) on the right side of Fig 3. This value is calculated based on the distance between trains *T*_*n*−1_ and *T*_*n*_—which can be measured by a sensor— and the average speed of train *T*_*n*_, considering the last *k* iterations as stipulated by Eq 2:

$$\begin{array}{c} ETNextTrain\left(t\right)=d\left(x_{n}\left(t\right),x_{n-1}\left(t\right)\right)/{\bar{v}}_{n}\left(t,k\right), \end{array}$$ (2)

where *d*(*x*_*n*_(*t*), *x*_*n*−1_(*t*)), is the Euclidean distance at time *t* between the position *x*_*n*−1_ of the train located at the station and the position *x*_*n*_ of the next train *T*_*n*−1_; ${\bar{v}}_{n}\left(t,k\right)$ is the average speed of the train *T*_*n*_ during the last *k* iterations in function of time *t*. When the anti-pheromone value is more than or equal to *ETNextTrain*, the condition to depart is accomplished, then the train leaves the station and the anti-pheromone value is reset. The balance between these two variables makes it possible that the train preserves and restores the headway in a decentralized way using local information, but can also adapt to changes in the system. The behavior can be summarized in two main situations: 1. When the anti-pheromone value is high and the *ETNextTrain* is low, this indicates that the train ahead departed long ago and the train behind will arrive soon; for this reason, the train in the station can depart. 2. If the anti-pheromone value is low and the *ETNextTrain* is high, the train in the station will have to wait until the next train is closer or the previous train is farther. The video https://youtu.be/1AS9dWdr6kk shows a headway regulation experiment using SOM-II. It starts with a configuration of the trains accumulated one behind the other and ends up with almost equal headways. Fig 5 displays the NetLogo simulator along with signs and graphics to observe the system performance. The reader can access the simulator and source code at https://github.com/gcarreon/pts-simulator.

### Extended Gipps model

The simulation uses an extension of Gipps’ car-following model [25] to characterize the train flow with three main external adjustments: 1. A line of vision that allows the train identify the agent ahead (i.e., trains or traffic lights); 2. A safe distance between trains and; 3. An autonomous brake using the “phantom object” strategy [26]. The model considers discrete time and continuous space. The equation to calculate the speed at time *t* + *τ* is:

$$\begin{array}{c} u_{n}\left(t+\tau \right)=min\left\{G_{a}\left(t\right),G_{d}\left(t\right)\right\}, \end{array}$$ (3)

$$\begin{array}{c} G_{a}\left(t\right)=u_{n}\left(t\right)+2.5a_{n}\tau \left(1-u_{n}\left(t\right)/V_{n}\right)\sqrt{0.025+u_{n}\left(t\right)/V_{n}}, \end{array}$$ (4)

$$\begin{array}{c} G_{d}\left(t\right)=b_{n}\tau +\sqrt{{b_{n}}^{2}\tau^{2}-b_{n}\left(2\left(x_{n-1}\left(t\right)-s_{n-1}-x_{n}\left(t\right)\right)-u_{n}\left(t\right)\tau -u_{n-1}{\left(t\right)}^{2}/\hat{b}\right)}, \end{array}$$ (5)

where *a*_*n*_ is the maximum acceleration which the driver of vehicle *n* wishes to undertake; *b*_*n*_ is the most severe braking event that the driver of vehicle *n* can make (*b*_*n*_ < 0); *s*_*n*_ is the effective size of vehicle *n*, *V*_*n*_ is the desired speed of vehicle *n*, *x*_*n*_(*t*) is the location of the front of vehicle *n* at time *t*, $\hat{b}$ is the value of *b*_*n*−1_ estimated by the driver of vehicle *n* which cannot be known from direct observation; *u*_*n*_(*t*) is the speed vehicle *n* at time *t*, and *τ* is the apparent reaction time which is constant for all vehicles.

Eqs 4 and 5 represent the acceleration and deceleration respectively, which are compared to update the new speed *u*_*n*_(*t* + *τ*) according to the distance of the object in front. The update of the position of vehicle *n* at time *t* + *τ* is,

$$\begin{array}{c} x_{n}\left(t+\tau \right)=x_{n}\left(t\right)+0.5\left(u_{n}\left(t\right)+u_{n}\left(t+\tau \right)\right)\tau . \end{array}$$ (6)

The reaction time of the driver described by *τ*, is commonly set at 2/3 of a second. The simulation tick’s value is taken form this parameter. The physical characteristics of the train flow are described directly by the parameters of the model; this allows to set the values that are empirically obtained.

### Extension 1: Line of vision

**Fig 6:**
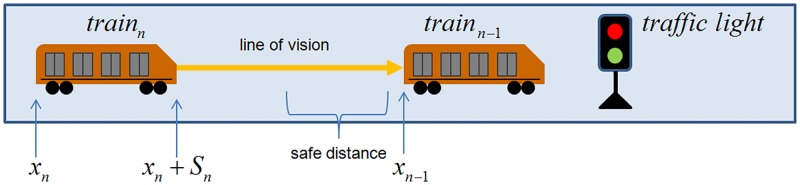
Agents in the line of vision. The line of vision value is set to 10 patch units (i.e. 1500 meters). This is an efficient strategy because in most cases is not necessary compute the acceleration (Eq 4) and deceleration (Eq 5) in each step of the simulation.

A new parameter, called line of vision, allows the train to identify the trains or the traffic lights ahead of it. If the agent is within the line of vision, the distance is calculated to determine the behavior of the train (Fig 6). For instance, if there is a train ahead, Eq 3 is executed; if there is a traffic light in green or red a Eqs 4 or 5 is computed respectively. And if there are both in the line of vision then the minimum distance to the agent is considered. If no agent is found, then Eq 4 is computed.

### Extension 2: Safe distance

A minimum safe distance was defined; this is a fundamental operational rule in PTS, so trains do not set too close one behind the other, as in the original model. The expression to calculate the distance is part of the deceleration equation (Ec.5), which becomes (Ec.7), as follows:

$$\begin{array}{c} x_{n-1}-\left(safedistance+S_{n}+x_{n}\right). \end{array}$$ (7)

The safe distance can be seen as an increment of the effective size of the train *S*_*n*_ to model the space between trains. It remains constant for all trains and the value of the parameter is equal to the train length, *i.e.* 150 meters.

### Extension 3: Autonomous braking

**Fig 7:**
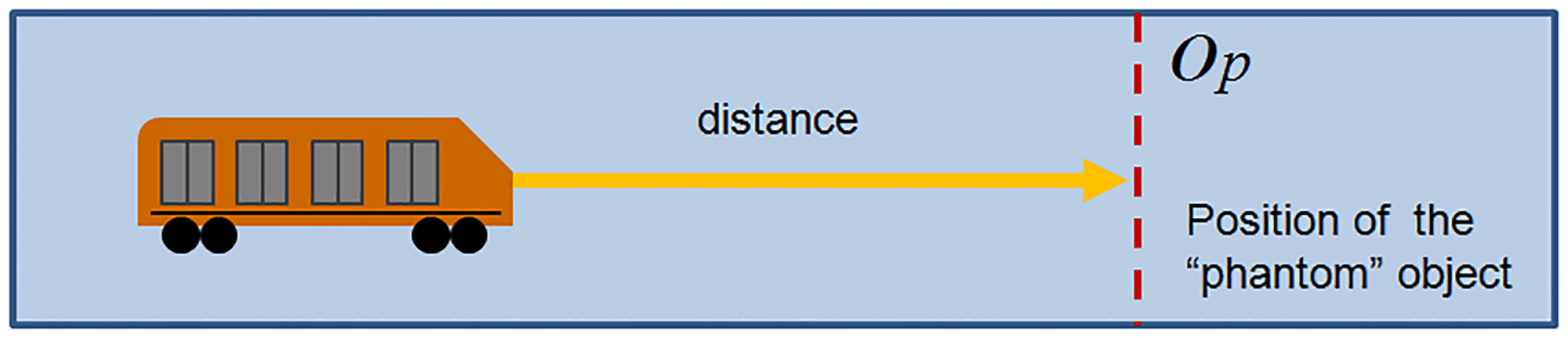
“Phantom strategy” with optimal position. To set the correct position of the “phantom” object considering the train speed, a table of calculated distances are loaded *a priori*.

Autonomous braking using the “phantom object” strategy is used without modifying the original Gipps’ Model. An object of size zero is placed at position *x*_*n*−1_ and time *t* in the optimal distance for the train to begin braking immediately. To find the correct position, a systematic and exhaustive search in the parameter space of *V* (desired speed) and *b* (brake) was performed. The elapsed time and distance before the train stops completely were measured (Fig 7). This functionality allows braking at any time and, as a consequence, offers the possibility of decreasing the desired speed by braking and then setting the new *V* when this value is reached. This extension was used to simulate possible mechanical failures of specific trains, for example, reducing the train’s desired speed to zero to test how the regulatory methods react under these circumstances.

Parameter values of acceleration and braking were optimized to represent the dynamical properties of MXM trains adequately through an exhaustive and systematic parameter search. Maximum speed, estimated braking of the train ahead, security distance, vehicle size, line of vision, safe distance and reaction time of driver were set according to appropriate realistic values. Table 1 resumes this information.

**Table 1:** Parameters used in Gipps’ model.

Description	Parameters value
Reaction time	*τ* = 2/3*sec*
Train size	*S*_*n*_ = 150*m*
Safe distance	*safedistance* = 150*m*
Line of vision	*lineofvision* = 1500*m*
Maximum speed	*V*_*n*_ = 13*m*/*sec*
Acceleration	*a*_*n*_ = 3.8*m*/*sec*^2^
Deceleration	*b*_*n*_ = −1.15*m*/*sec*^2^
Estimated brake of train ahead	$\hat{b}=min\left(-3.0,\left(b_{n}-3.0\right)/2\right)m/sec^{2}$

The parameters considering the properties of MXM trains and infrastructure. The estimated brake of train ahead is taken from the original Gipps’ model.

## Results

We used the profile of the Line 1 (pink) of the MXM to test the performance of the regulatory methods, considering realistic distances, velocities, number of trains, and passenger demands. In the computer simulation, time is measured by *ticks*, where each tick corresponds to 2/3 of a second. The spatial unit is called *patch*, where each patch represents 150 meters. The time for updating Gipps’ model and the regulatory methods, and the length of the train, coincides with the value of the ticks and the size of the patch respectively. The values of the parameters of Gipps’ model, such as the speed, the acceleration, and deceleration, were established from a systematic and exhaustive search in the parameter’s space using the values that better adjusted to mechanical properties of the trains.

A cyclic line with the following elements was built: a railroad line of 19.5 kilometers; 20 stations with length of 150 meters distributed with the real inter-station segments of Line 1, (from Pantitlán station to Observatorio station); 16 trains equidistantly distributed along the line and traffic lights at each station. Each passenger’s travel was modeled with probabilities *P*_*arrival*_*k*__ and *P*_*departure*_*k*__ of arriving and departing at station *k* respectively. The set of all passenger’s travel models origin-destination flows. A sample of the average flow per station in the MXM contributed to define these probabilities. Intervals among arrivals of passengers at the station followed a Poisson distribution with an average of *λ*_*p*_ ticks.

Low (e.g. *λ*_*p*_ = 1) and high (e.g. *λ*_*p*_ = 20) values of *λ*_*p*_ model a high and low number of arrivals per period of time respectively. For example, if *λ*_*p*_ = 5, a new passenger arrives at the *k* station every 5/*P*_*arrival*_*k*__ ticks on average. Trains consist of one car with capacity of 180 passengers and only one door, as the results are scalable up to the 36 doors in the real train (four per each of nine wagons). According to empirical observations, the time of alighting and boarding is proportional to 1 passenger per second per door on average.

**Fig 8:**
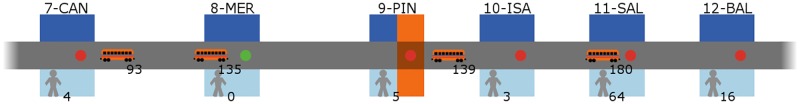
Fragment of the computer simulation of the Line 1 of the MXM. Stations are marked in blue; traffic lights are circles in red or green; trains show the number of current passenger load, and a person shows the number of passengers waiting in the station. The position of the point of measurement of the headway is marked in orange and shows where the empirical measurements were taken.

The GM considers three parameters: the minimum and maximum waiting time of the train at the station *T*_*min*_ and *T*_*max*_ respectively, and a Poisson distribution with mean *λ*_*r*_ that represents the delay due to passengers or caused by a late departure of the train. A point of measurement illustrated with orange mark on the line (Fig 8) was located to generate frequency histograms of time intervals between trains at such location (Pino Suárez station), that we called the *headway distributions*.

**Fig 9:**
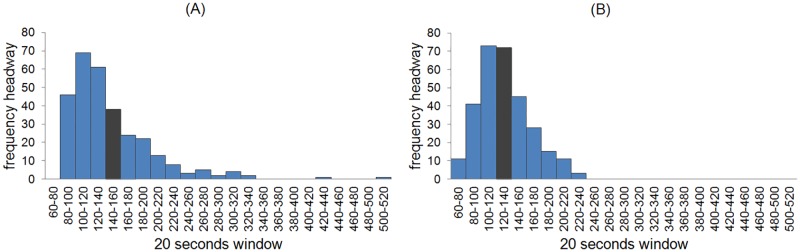
Histograms of the headway frequencies. The mean headway value in the real system and the GM simulation are (A) 147 and (B) 132 seconds, illustrated with a gray bar.

The headway distribution was obtained through 299 data measurements (Fig 9A) obtained from 12 hours of video recordings during high attendance or peak hours. Due to the instability of the phenomenon and in order to highlight the qualitative properties over the quantitative ones, we used time windows of 20 seconds in the histogram.

A systematic and exhaustive search over space *T*_*min*_, *T*_*max*_, and *λ*_*r*_ took place to find the values that better adjust to the real headway distribution. The ranges of the search were established from empirical measurements, considering a minimum and maximum waiting times in seconds and the delay in departure, which are 16 ⩽ *T*_*min*_ ⩽ 40, 20 ⩽ *T*_*max*_ ⩽ 200 and *λ*_*r*_ = {3, 6} respectively. For each possible vector (*T*_*min*_, *T*_*max*_, *λ*_*r*_) we ran the simulation until the headway distributions with 299 headway measurements was obtained. The Pearson correlation coefficient was subsequently calculated. The optimum values are: *T*_*min*_ = 24, *T*_*max*_ = 80, and *λ*_*r*_ = 3, which reached a maximum Pearson correlation coefficient of 0.98 (Fig 9B, see below). In the real system, the headway may be exceptionally longer than 7 minutes due to mechanic failures; however, in the simulation the maximum headway was about 4 minutes.

**Fig 10:**
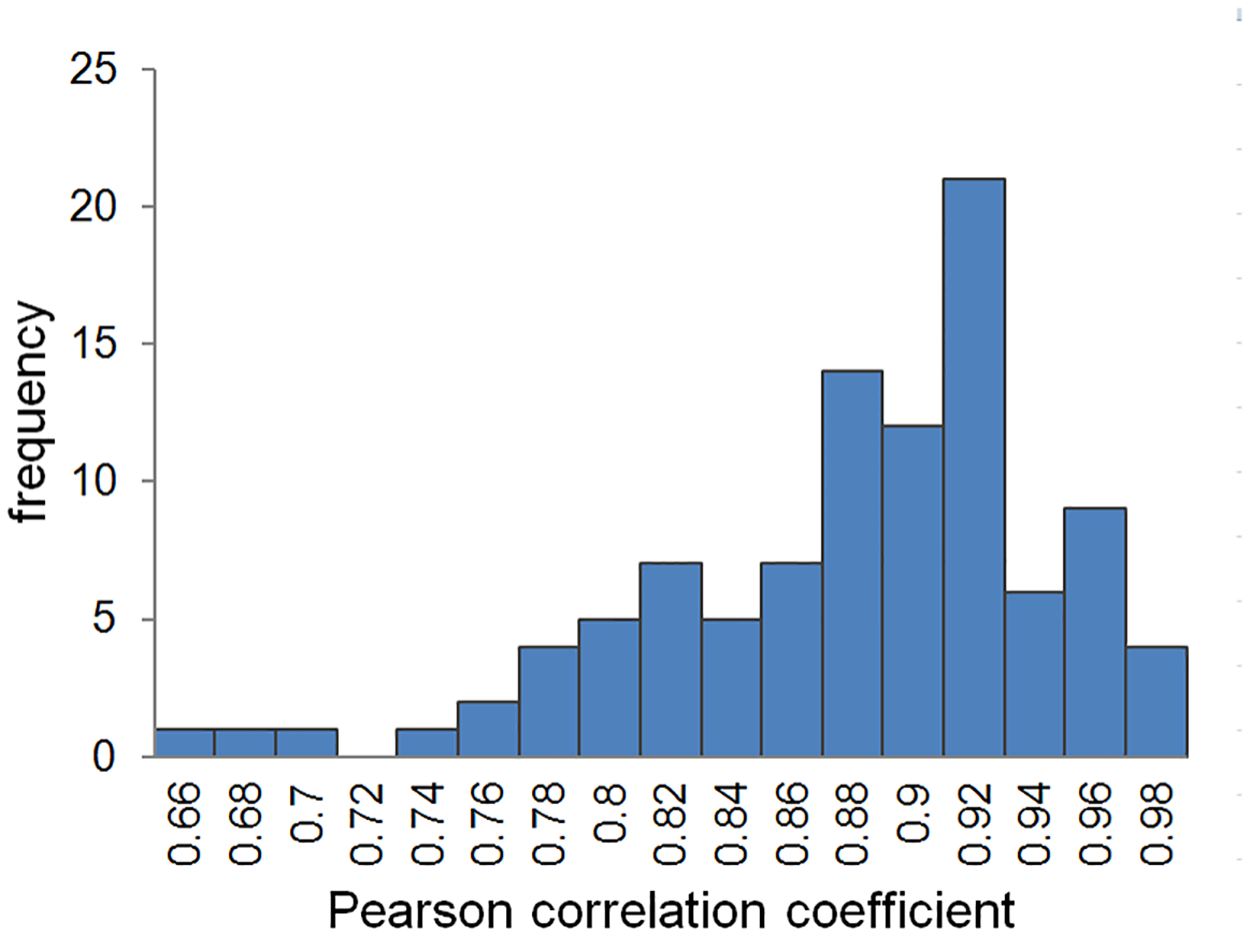
Histogram frequency of the Pearson correlation coefficient between the headway distribution data of Line 1 and 100 GM simulations.

The initial conditions considered empty trains and stations and trains placed equidistantly along the line. The simulation was run a hundred times with the best set of parameters (*T*_*min*_ = 24, *T*_*max*_ = 200, *λ*_*r*_ = 3) and different random seeds to observe statistical variations of Pearson’s correlation coefficient between GM simulations and the real headway. The histogram (Fig 10) shows that the maximum correlation value is 0.98 and the mean is 0.87 with standard deviation 0.06 for the 100 experiments. The statistical variation is produced by the probability distribution of the arriving and departing of passengers in the stations and the delay in the departure of the trains.

**Fig 11:**
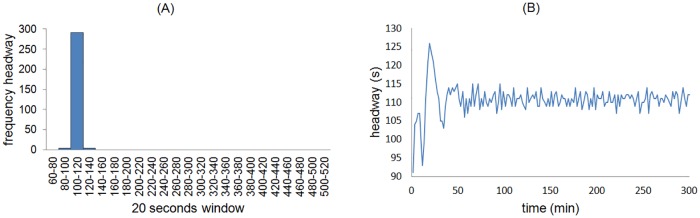
Behavior of the headway with SOM-II. (A) Distribution of the headway frequencies, keeping the same scale of windows size to compare adequately with MXM Line 1 and GM distribution headway. (B) A typical time series of the headway measured at the simulated Pino Suárez station.

The performance of the SOM-II substantially improves the headway stability (Fig 11A) in relation to GM (Fig 9B). The histogram of the headway frequencies shows a mean of 110 seconds, which are 5 to 10 seconds less than the theoretical optimal headway, which is 115-120 seconds for Line 1 in low demand conditions according to the operational personnel (although there is not official document sustaining such opinions); therefore, the inequality of Eq 1 holds. The SOM-II adapts to local changes produced by the influx of passengers. The time series of the headway shows high fluctuations at the initial values but the trains adapt to a supra-optimal configuration after about 40 to 50 minutes (Fig 11B). The small differences in headway values are produced by the passenger influx variation.

**Fig 12:**
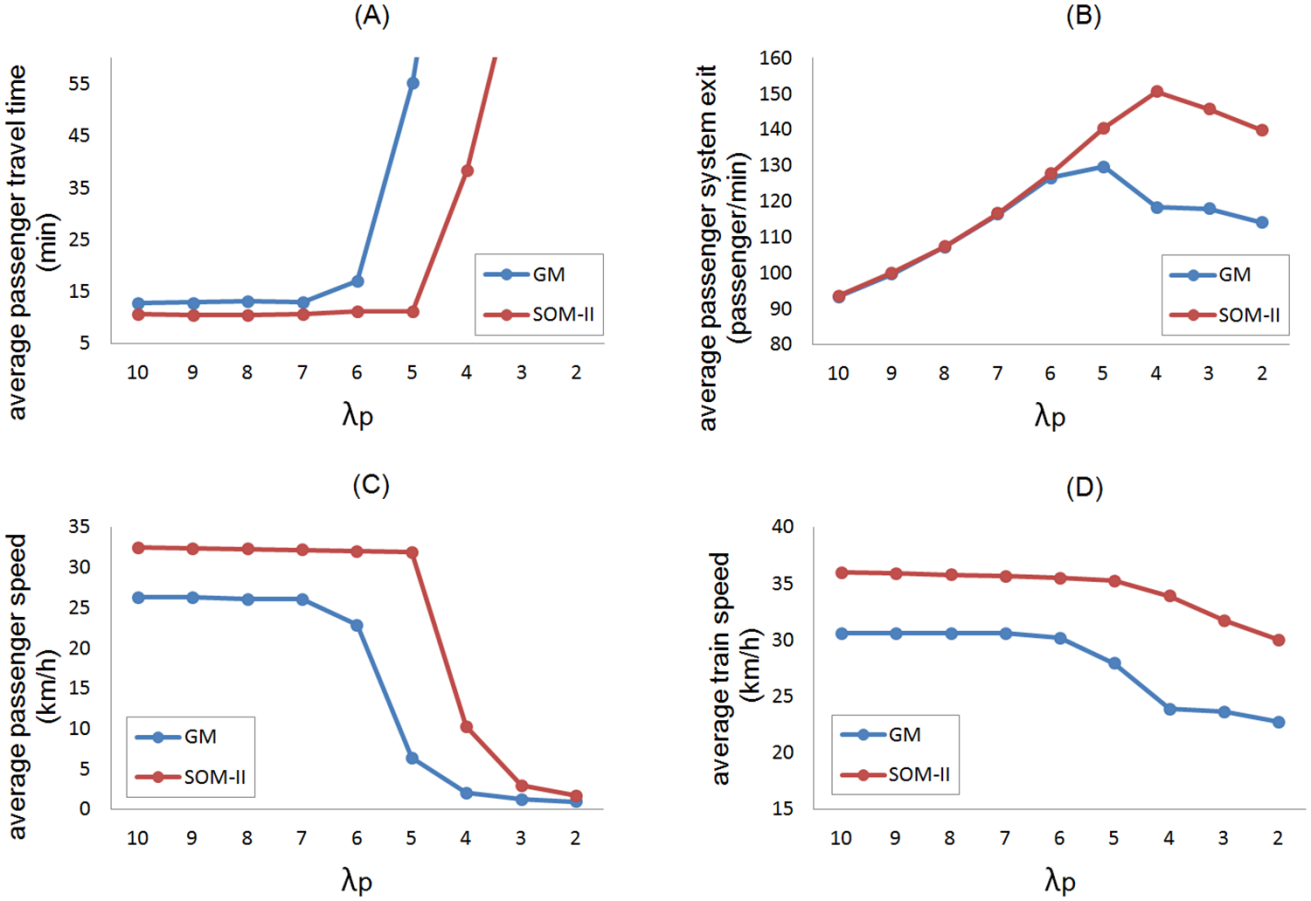
Comparison of the methods. (A) Average passenger travel time, (B) average passenger system exit, (C) average passenger speed, (D) average train speed. In graph (A) values of *λ*_*p*_ = {2, 3} are not included because the time increment exponential due to overcrowding.

The performance of SOM-II and GM can be compared with averaged measures; we consider passengers’ travel time, passenger’ exit flow, passenger speed, and train speed. The control parameter is the passenger’s influx *λ*_*p*_ as defined at the beginning of the section and varied in the interval 2 ⩽ *λ*_*p*_ ⩽ 10. For each *λ*_*p*_ value the simulation was run for 100,000 iterations; to discard transient dynamics, the first 30,000 iterations were not considered. To keep consistency with the previous results, the same parameters of both regulatory methods and the Gipps’ model were used. Fig 12 shows the results of the performance.

The GM decrease its performance when *λ*_*p*_ < 7 (Fig 12A), since the average passengers’ travel time starts increasing due to the system’s saturation. For example the first and second station in the line, Pantitlán and Zaragoza, have the highest probability of passenger arrival, but the overcrowding phenomenon starts in the stations Salto del Agua and Balderas, positions 11 and 12 respectively over the line and then impact negatively to other stations. The pattern of propagation of station overcrowding depends of the alighting and boarding probabilities at each station. The average speed of passengers (Fig 12C) and the average speed of trains decreases (Fig 12D) when *λ*_*p*_ = 6; the performance of the SOM-II decreases for *λ*_*p*_ < 5 (Fig 12A, 12C and 12D), which implies a capacity increase of the transportation system through headway regulation in average 20% for *λ*_*p*_ < 5 (Fig 12B).

**Fig 13:**
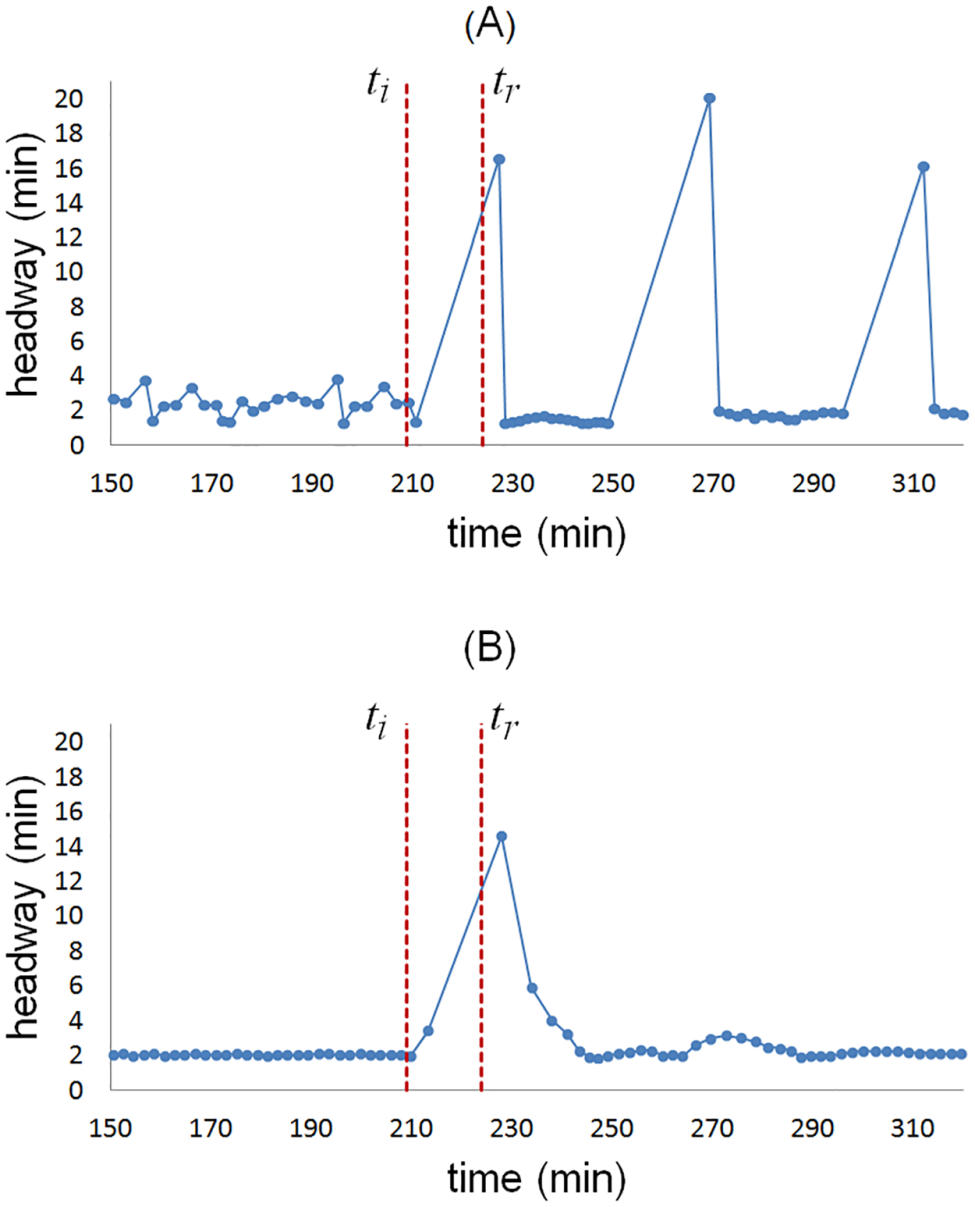
Modeling a mechanical failure at a specific time. The mechanical failure begins and ends in the minutes *t*_*i*_ = 209 and *t*_*f*_ = 224, respectively. (A) in GM series, the high values of the headway represents the formation of clusters. (B) The SOM-II time series is robust against the perturbation and is capable of restoring the system in a short time.

**Fig 14:**
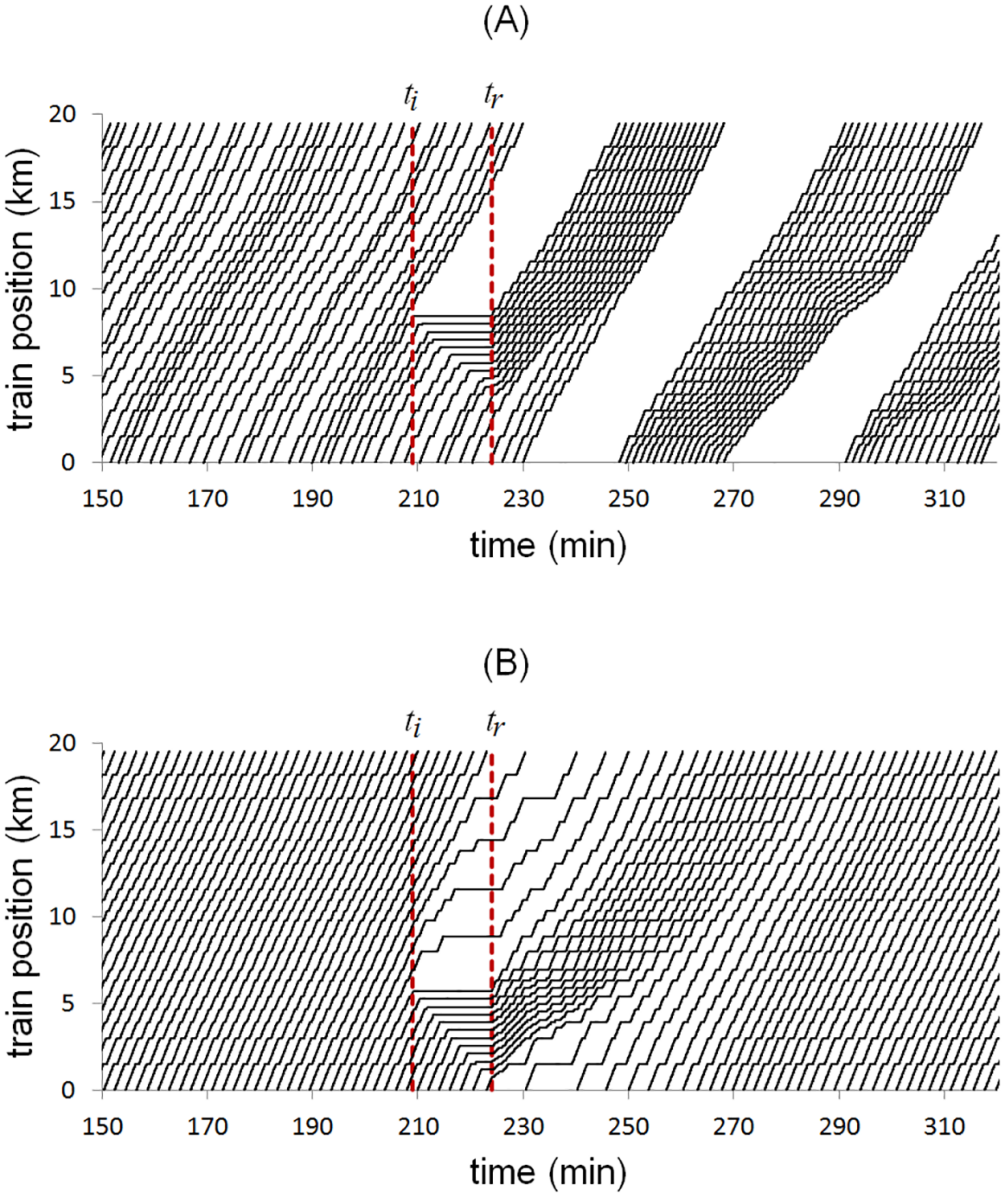
Time-space diagram of the trains. (A) After the mechanical failure in GM, the system exhibits a striped pattern characteristic of the equal headway instability. (B) The SOM-II has a homogeneous pattern and stable before and after the mechanical failure, the trains in front of *train*_0_ already wait more at stations even before the failure ends, since the balance between the variables *ETNextTrain* and *antipheromoneStation* delay the departure. This improves the resilience and accelerates the recovery of the system.

In order to test the headway resilience capacity of GM and SOM-II, the following experiment was devised: the system with initial condition as mentioned before was interrupted in the minute 209 by stopping the *train*_0_ for 15 minutes before the headway measure point, the stop time interval start and finalize with *t*_*i*_ and *t*_*r*_ respectively, shown with red dashed lines in Figs 13 and 14, which resulted in the formation of a train cluster of size 10 with a safe distance of 300 meters between them, respecting an operational rule in the MXM (Fig 14).

After that, the stopped train restarts its flow. On the one hand, the time series of GM shows that the headway cannot restore its previous dynamics as a convoy of aggregated trains is formed with interval separation of 16 to 20 minutes and the system needs external intervention to recover; the time spent by the *train*_0_ to arrive at the headway measuring point is 16.59 minutes (depicted by the first peak in Fig 13A), while the minimum headway value is 1.28 minutes due to the main cluster formation (Fig 14A).

The passenger accumulation is increased at stations due to the service interruption. The accumulating trains can stopped at stations or between them. In the first case, the dwell time can exceed the maximum waiting time at stations *T*_*max*_. Still, passengers can board the train until its maximum capacity is reached or the service is restored.

On the other hand, the time series of SOM-II shows a rapid recovery of the headway; at the restoration of the service, a gap of 14.60 minutes is formed, but decreases until it recovers by itself completely in about 50 minutes (Fig 13B), the mean value of the headway is 2.4 minutes with standard deviation 0.72 and minimum value 1.84 minutes, which are values within the operational range.

## Discussion

This paper presented two methods to regulate public transportation systems; the General method (GM) and the Self-Organizing II method (SOM-II). The results of GM correlated well with the headway data collected empirically in the MXM. Calibrating and validating our computational methods in this way, we can argue that results of SOM-II are reliable. We can expect that in case of implementing this method in a real system, an improvement in average journey time of approximately 20% and a decrease of 25% in mean headway values, as well as the ability to maintain and recover regular headways.

The GM models the headway distribution of MXM based on minimum and maximum waiting time of train at stations and a delay component *λ*_*r*_. A Poisson distribution with mean *λ*_*p*_ represents the time between passenger arrivals in the system. The appropriate values of the parameter’s simulation produce a high correlation with the empirical measurements carried out in the MXM. The model shows that the system has a low performance due to an inadequate regulation of the headway. The factors that influence the headway instability are the station overcrowding, which affects the boarding and alighting time, and the time departure of the train. To isolate the components that promote delays and analyze the behavior of the headway, we explored a scenario without delay and a low influx of passengers; that is for example, *λ*_*r*_ = 0 and *λ*_*p*_ > 10; the time average for a journey improved to 10%. However, the headway values reflect a poor performance of the system.

The system regulated with GM exceeds its capacity when *λ*_*p*_ ≤ 6 (high influx of passengers). In this condition, the performance decreases and trains cluster. This characteristic is also observed within other models [10, 31, 32]. On the contrary, the SOM-II shows a superior performance, seemingly better that the theoretical optimum [21]. It was also shown that the headway is recovered and preserved under any initial distribution of trains, even in a cluster configuration. With values of a high saturation of passengers such as *λ*_*p*_ = 5, the headway remains stable and the system capacity is increased, compared with GM (Fig 12A).

The SOM-II eliminates effectively the equal headway instability, *i.e.* it reduces bus-bunching by 100%, adaptively adjusting the waiting time at stations. For *λ*_*p*_ ≥ 7, SOM-II improves over GM the average travel time of passengers by 18%. For *λ*_*p*_ = 6, the improvement is of 34%; for *λ*_*p*_ = 5, 79%; while the improvement is of 67% for *λ*_*p*_ = 4. Overcrowding occurs with GM for *λ*_*p*_ ≤ 6 and with SOM-II for *λ*_*p*_ ≤ 4. The system collapses with GM for *λ*_*p*_ ≤ 5 and with SOM-II for *λ*_*p*_ ≤ 3. The present results can be compared with the the work presented in [15] in which online learning and simulation approach showed a bus-bunching reduction rate of 65% and a reduction of waiting time at stations of about 4.5%. As future work, our proposal could be combined with a stop-skipping scheme [14]to reduce overcrowding at stations.

Computer simulations are useful for studying public transportation systems, as one can: 1. Explore different scenarios; for instance, to accurately simulate failures of trains, to use functions for the probability of arrival of passengers or implement strategies of dosage of empty trains to serve overcrowded stations; 2. Estimate the stability of the headway from initial conditions that along with real time information systems allows performance estimations all day, and 3. Scale and model the complete PTS, for example, to analyze the dynamics of transfer stations. This model considers parameters and relevant processes to regulate the headway and influence the real phenomenon, although it is not the intention to give a detailed description of all processes and variables that participate in PTS dynamics.

### The Mexico City Metro

The delay parameter *λ*_*r*_ considered in the simulation represents three observed dynamics in MXM: 1. the possible delay in the process of boarding and alighting; 2. the delay caused by closing doors due to saturation and inappropriate passenger behavior; and, 3. Delays in the departure of the trains possibly due to train’s cluster formation. The first two points can be dealt with an adequate boarding strategy to guide passenger behavior; while the third can be regulated implementing SOM-II.

**Fig 15:**
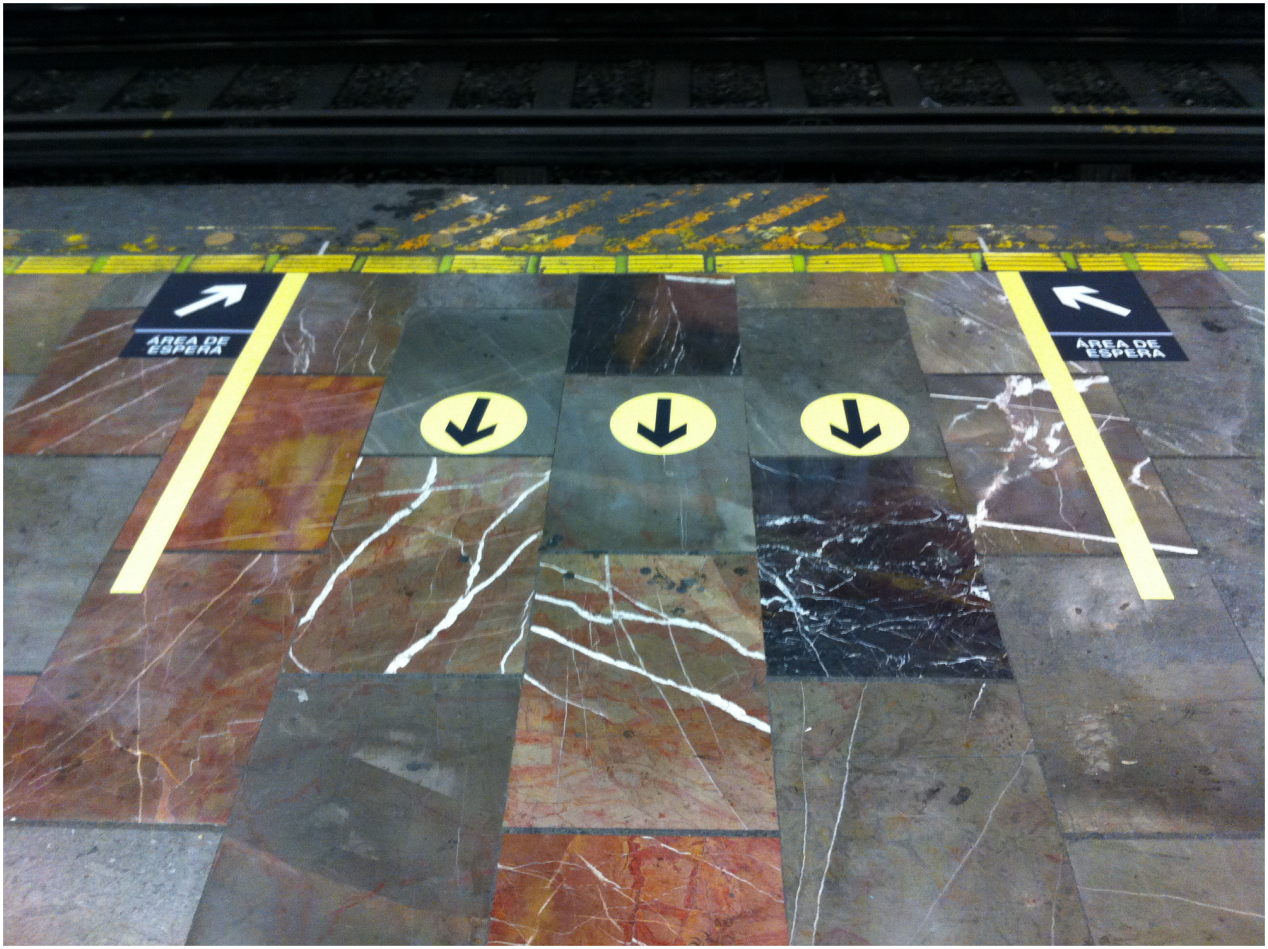
Guide sign on the platform. The components of the guide signs are: three circles that indicate the way to exit, two vertical lines dividing the waiting area, and two black boxes with sideline arrows to indicate the boarding orientation.

The ideas considered in this work and the empirical observations and measurements encouraged the development of a pilot experiment, installed on December 4th, 2016 at Balderas station of Line 1 of the MXM. which consisted of placing guide signs on the platform (Fig 15) to regulate the flows of boarding and alighting of passengers and minimize the dwell time. We considered the following components of the dwell time: the time of alighting and boarding of passengers; the remaining time of the open doors when the passenger can still alight or board; and the delay in the door closing process which represents a significant delay during overcrowding. In the MXM under normal conditions, the door closing process takes two or tree seconds, while in overcrowding conditions it can reach 55 seconds due to passenger behavior.

The signs allow users to identify the position of the doors before a train arrives at the station and wait in zones to avoid any obstruction of alighting passengers.

**Fig 16:**
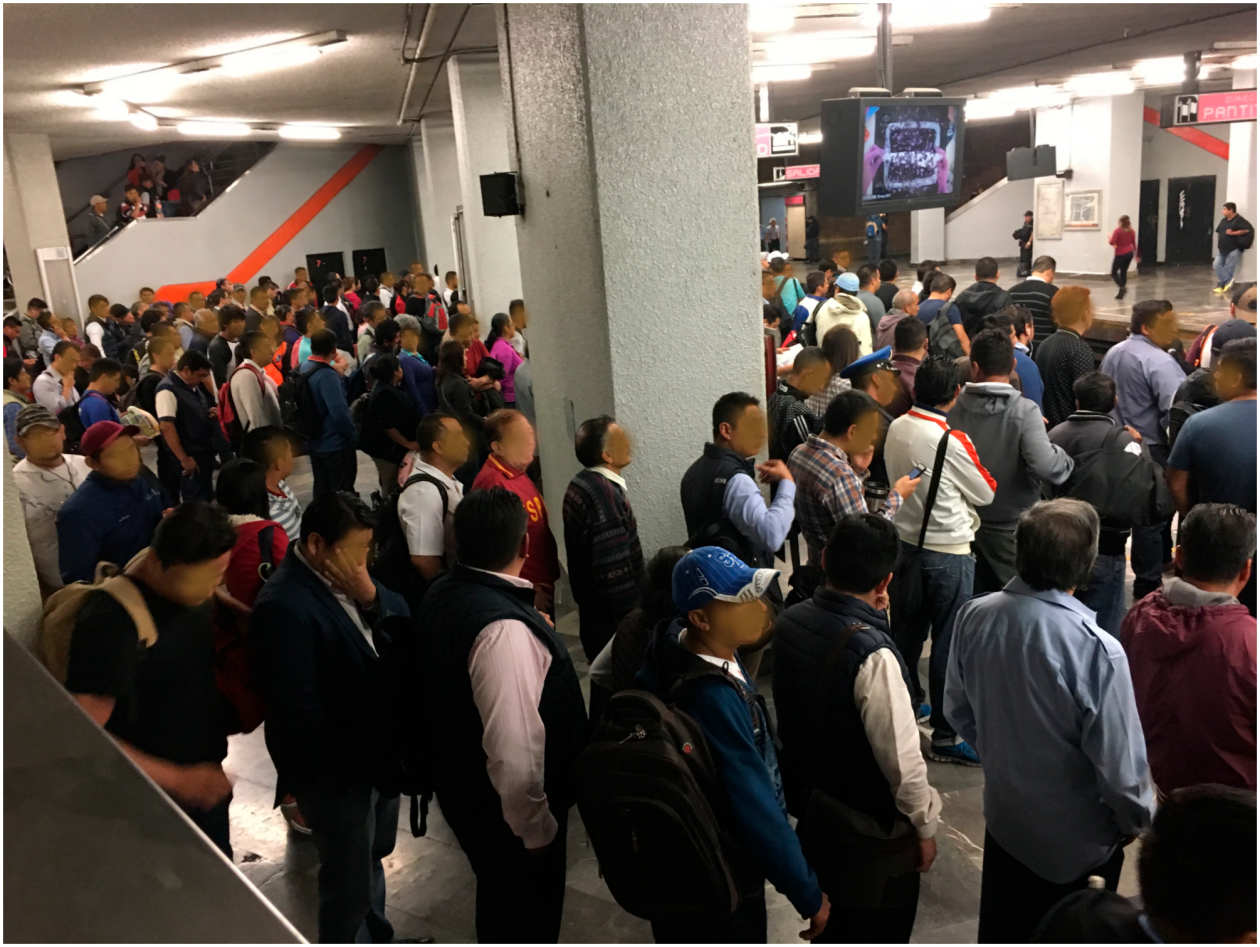
Regulatory strategy in operation. Passengers wait in the line the train arrival and the alighting area remains clear.

Users gathered at any of the two waiting zones, and when the train arrives passengers alight without any obstruction; subsequently, users waiting at the platform can board in two simultaneous flows, and those who cannot achieve it will remain at the waiting zone (Fig 16).

The following are the results of the study: 1. Conflicts and pushes during the boarding and alighting were eliminated almost entirely; 2. The time of boarding and alighting reduced about 10 to 15%; 3. The obstructions when closing the doors were reduced about 15 to 25%, and 4. Users positively adopted the regulation strategy.

We expected that passengers would group into clusters to board the train; however, they spontaneously formed a lines (Fig 16). This increased order and consequently a significant reduction of conflicts and pushes. This “first come, first board” dynamic was disseminated and became an accepted rule. The regulatory method was satisfactorily implemented, and because of this, it has been spread to other Metro stations. The method accelerated the boarding and alighting of passengers in high influx stations, such as transfer stations. This new low-cost-high-impact scheme shows a real benefit on the quality of travel and is generating a change in the passenger culture. The reader is invited to see the videos of the project at the URLs https://youtu.be/eoXsvYQ8H_s and https://youtu.be/kshOlqJU3Hs.

### Recommendations

These are suggestions to improve the performance of PTS.

- Place suitable guide signs on the platform. With this information, users will move correctly and will be sure about the position of the doors and avoid obstructions for alighting passengers. Caracas, Río de Janeiro, Dubai, Hong Kong, Singapur, Tokio, Seoul, Beijing, Montreal, Munich, Boston, Mexico City, and other cities currently have schemes of signs on the platform.
- Enable real time information systems about the time of waiting and available space on trains, which would make easier for users to decide the alternative routes to use in case of saturation. There are mobile applications that could help with this task.
- Raise awareness among passengers through information campaigns to promote a proper use of the services and the implications of bad behavior, and also to explain basic concepts of operation, such as the equal headway instability and the slower-is-faster effect [22].
- Use a computer simulation to short-term predict critical situations, to prevent system collapse, and take appropriate actions.
- Design methods to control and regulate headways. The SOM-II is feasible for its implementation as it would substantially improve the service and would help to increase the thresholds of a system collapse about a 25%.

### Conclusion

The headway distribution was considered as an indicator to measure the performance of public transportation systems. The Self-Organizing Method II shows that with appropriate local interaction rules, it is possible to regulate the headway globally and adaptively. The method uses variables with an explicit representation of the real phenomenon, which indicates a guide for the technological implementation and its possible regulation. In addition, we consider that the passenger dynamics directly impact the achievement of the service. The pilot experiment carried out in the Mexico City Metro showed that a change of interaction rules among passengers can improve the system considerably. The local and global interaction mediators allow reducing delays and an adaptive service regulation.

## References

[1] World metro database;. Available from: http://mic-ro.com/metro/table.html.

[2] Sistema de Transporte Colectivo Metro;. Available from: http://www.metro.cdmx.gob.mx/operacion/cifras-de-operacion.

[3] Pagels HR. The Dreams of Reason: The Computer and the Rise of the Sciences of Complexity. Bantam Books; 1989.

[4] Gershenson C, editor. Complexity: 5 Questions. Automatic Peess / VIP; 2008 Available from: http://tinyurl.com/ovg3jn.

[5] Gershenson C. The Implications of Interactions for Science and Philosophy. Foundations of Science. 2013;18(4):781–790. 10.1007/s10699-012-9305-8

[6] Louf R, Roth C, Barthelemy M. Scaling in Transportation Networks. PLOS ONE. 2014;9(7):1–8. 10.1371/journal.pone.0102007 PMC4100765 25029528

[7] Moreira-Matias L, Mendes-Moreira J, de Sousa JF, Gama J. Improving Mass Transit Operations by Using AVL-Based Systems: A Survey. IEEE Transactions on Intelligent Transportation Systems. 2015;16(4):1636–1653. 10.1109/TITS.2014.2376772

[8] Ceder A. Public Transit Planning and Operation. Taylor & Francis; 2007 Available from: https://books.google.com.mx/books?id=QucV7bDg9N4C.

[9] Welding PI. The Instability of a Close-Interval Service. OR. 1957;8(3):133–142. 10.2307/3007157

[10] Gershenson C, Pineda LA. Why does public transport not arrive on time? The pervasiveness of equal headway instability. PLoS ONE. 2009;4(10):e7292 10.1371/journal.pone.0007292 19862321 PMC2762539

[11] Daganzo CF. A headway-based approach to eliminate bus bunching: Systematic analysis and comparisons. Transportation Research Part B: Methodological. 2009;43(10):913–921. 10.1016/j.trb.2009.04.002.

[12] Lin Tm, Wilson NH. Dwell time relationships for light rail systems. Transportation Research Record. 1992;(1361):287–295.

[13] Bartholdi JJ, Eisenstein DD. A self-coördinating bus route to resist bus bunching. Transportation Research Part B: Methodological. 2012;46(4):481–491. 10.1016/j.trb.2011.11.001.

[14] Liu Z, Yan Y, Qu X, Zhang Y. Bus stop-skipping scheme with random travel time. Transportation Research Part C: Emerging Technologies. 2013;35(Supplement C):46–56. 10.1016/j.trc.2013.06.004.

[15] Moreira-Matias L, Cats O, Gama J, Mendes-Moreira J, de Sousa JF. An online learning approach to eliminate Bus Bunching in real-time. Applied Soft Computing. 2016;47(Supplement C):460–482. 10.1016/j.asoc.2016.06.031.

[16] Yu H, Chen D, Wu Z, Ma X, Wang Y. Headway-based bus bunching prediction using transit smart card data. Transportation Research Part C: Emerging Technologies. 2016;72(Supplement C):45–59. 10.1016/j.trc.2016.09.007.

[17] Yu H, Wu Z, Chen D, Ma X. Probabilistic Prediction of Bus Headway Using Relevance Vector Machine Regression. IEEE Transactions on Intelligent Transportation Systems. 2017;18(7):1772–1781. 10.1109/TITS.2016.2620483

[18] Li Y, Wang X, Sun S, Ma X, Lu G. Forecasting short-term subway passenger flow under special events scenarios using multiscale radial basis function networks. Transportation Research Part C: Emerging Technologies. 2017;77(Supplement C):306–328. 10.1016/j.trc.2017.02.005.

[19] Liang S, Zhao S, Lu C, Ma M. A self-adaptive method to equalize headways: Numerical analysis and comparison. Transportation Research Part B: Methodological. 2016;87:33–43. 10.1016/j.trb.2016.02.008.

[20] Daganzo CF, Pilachowski J. Reducing bunching with bus-to-bus cooperation. Transportation Research Part B: Methodological. 2011;45(1):267–277. 10.1016/j.trb.2010.06.005.

[21] Gershenson C. Self-organization leads to supraoptimal performance in public transportation systems. PLoS ONE. 2011;6(6):e21469 10.1371/journal.pone.0021469 21738674 PMC3127858

[22] Gershenson C, Helbing D. When slower is faster. Complexity. 2015;21(2):9–15. 10.1002/cplx.21736

[23] Bonabeau E. Agent-based modeling: Methods and techniques for simulating human systems. Proceedings of the National Academy of Sciences of the United States of America. 2002;99(Suppl 3):7280–7287. 10.1073/pnas.082080899 12011407 PMC128598

[24] Wilensky U. NetLogo; 1999. Available from: http://ccl.northwestern.edu/netlogo.

[25] Gipps PG. A behavioural car-following model for computer simulation. Transportation Research Part B: Methodological. 1981;15(2):105–111. 10.1016/0191-2615(81)90037-0.

[26] Spyropoulou I. SIMULATION USING GIPPS’ CAR-FOLLOWING MODEL—AN IN-DEPTH ANALYSIS. Transportmetrica. 2007;3(3):231–245. 10.1080/18128600708685675

[27] Fayyaz SK, Liu XC, Porter RJ. Genetic Algorithm and Regression-Based Model for Analyzing Fare Payment Structure and Transit Dwell Time. Transportation Research Record: Journal of the Transportation Research Board. 2016;2595:1–10. 10.3141/2595-01

[28] Milkovits M. Modeling the Factors Affecting Bus Stop Dwell Time: Use of Automatic Passenger Counting, Automatic Fare Counting, and Automatic Vehicle Location Data. Transportation Research Record: Journal of the Transportation Research Board. 2008;2072:125–130. 10.3141/2072-13

[29] Theraulaz G, Bonbeau E. A Brief History of Stigmergy. Artif Life. 1999;5(2):97–116. 10.1162/106454699568700 10633572

[30] Bonabeau E, Dorigo M, Theraulaz G. Swarm Intelligence: From Natural to Artificial Systems Santa Fe Institute Studies in the Sciences of Complexity. New York: Oxford University Press; 1999.

[31] Nagatani T. Fluctuation of tour time induced by interactions between cyclic trams. Physica A: Statistical Mechanics and its Applications. 2004;331(1):279–290. 10.1016/j.physa.2003.07.007.

[32] O’Loan OJ, Evans MR, Cates ME. Jamming transition in a homogeneous one-dimensional system: The bus route model. Phys Rev E. 1998;58:1404–1418. 10.1103/PhysRevE.58.1404

